# Effect of Argon Injection into the Down-Leg of RH on the Inclusion Removal in Industrial Trials

**DOI:** 10.3390/ma19020244

**Published:** 2026-01-07

**Authors:** Yukang Pan, Yanhui Sun, Yang He, Xiaodong Yang, Baohui Yuan, Jianhua Liu

**Affiliations:** 1 Collaborative Innovation Center of Steel Technology, University of Science and Technology Beijing, Beijing 100083, China; yukang_pan@163.com (Y.P.);; 2 Institute of Engineering Technology, University of Science and Technology Beijing, Beijing 100083, China; heyang2020@ustb.edu.cn; 3 Pangang Group Xichang Steel and Vanadium Co., Ltd., Steelmaking Plant, Xichang 615000, China; xiaodong2984@163.com; 4 State Key Laboratory of Vanadium and Titanium Resources Comprehenssive Utilization, Panzhihua 617000, China; yuanbaohui20@163.com

**Keywords:** argon injection, inclusion removal, bubble, RH refining

## Abstract

The novel industrial trial is conducted to investigate the effect of argon injection into the down-leg of the RH degasser on the inclusion removal. The ‘cold steel plate dipping’ is used to take samples of molten steel and argon bubbles from the RH ladle. The industrial CT detection and electron microscope observation are applied to analyze the bubble characteristics. The results show that the size of bubbles generated by argon injection in the down-leg ranges from 7 to 1430 μm. Among them, the number density of bubbles with a diameter of 60 μm is the largest, reaching 0.1 per mm^3^. After adopting the down-leg argon injection technology, the average oxygen activity at the end of the RH process decreases by 2.35 ppm, and the surface defects of cold-rolled sheets of all grades are reduced. Based on the theoretical analysis of bubble collision and adhesion to inclusions, the small-sized bubbles have a relatively high capture probability for inclusions smaller than 10 μm. Comprehensively analyzing the experimental results, it is found that the down-leg argon injection technology has an obvious effect on removing inclusions.

## 1. Introduction

Numerous alumina inclusions in ultra-low carbon steel can form nodules at the bottom of the stopper rod and on the inner wall of the nozzle, leading to blockage of the molten steel flow channel. This causes instability in the molten steel flow field within the nozzle and mold regions, resulting in fluctuations in mold level and slag entrainment [1,2,3,4]. Additionally, flow oscillations caused by the detachment of large nodules from the stopper rod and nozzle can also induce level fluctuations and slag entrainment. Moreover, detached large nodules and slag droplets resulting from slag entrainment can enter the mold, forming large, harmful inclusions that remain in the cast slab [5,6,7]. These inclusions can cause surface defects such as linear defects, peeling, pits, and cracks in cold-rolled sheets during subsequent rolling, thereby reducing cast slab yield [8,9,10,11,12]. To enhance cast slab yield and improve the surface quality of cold-rolled sheets, it is essential to effectively reduce inclusions and improve the cleanliness of molten steel [13,14,15,16].

However, in the IF steel production process, as one of the main processes to remove inclusions, the ability of RH refining to remove inclusions still needs to be improved. Zeng et al. [17] reported that during a 20 min RH refining period—from aluminum deoxidation to vacuum breaking—the inclusion number density decreases by 70%, but the remaining inclusions are generally smaller than 10 μm, making removal from molten steel challenging. Wang et al. [18] found that to further remove inclusions larger than 5 μm, the RH vacuum treatment must be extended to 33 min, which prolongs the overall refining cycle.

Compared to traditional refining methods, bubble metallurgy offers distinct advantages, including increasing the flotation velocity of fine inclusions, avoiding alterations to the molten steel composition through inert gases, and exhibiting compatibility with other control technologies [19,20,21,22]. Based on the principles of bubble metallurgy, Su et al. [23] injected argon at flow rates of 1.5~6.5 Nm^3^/h into the ladle shroud and analyzed the effectiveness of inclusion removal via argon blowing through the ladle shroud. The results indicated that this technique significantly reduced the size of inclusions 5~20 μm and entirely eliminated inclusions larger than 20 μm. Zhang et al. [21,24] investigated the bubble generation mechanism and bubble behavior of argon injection in RH down-leg, through water model experiments, numerical simulations, and theoretical analysis. It is concluded that argon gas injected into the down-leg is broken into bubbles measuring 1~3 mm due to erosion, and fine inclusions are effectively removed.

Generally, bubble metallurgy technology can enhance the cleanliness of molten steel. However, its industrial application remains limited. Among related technologies, argon injection in the RH down-leg predominantly remains at the laboratory or simulation stage. Therefore, this work implements down-leg argon injection (DLAI) technology during the industrial production of IF steel in a steel plant. The ‘cold steel plate dipping’ is employed to take a sample of molten steel and argon bubbles. In addition, the theoretical analysis of bubble collision and adhesion to inclusions is provided.

## 2. Experimental Methodology

### 2.1. Method of Down-Leg Argon Injection

**Figure 1:**
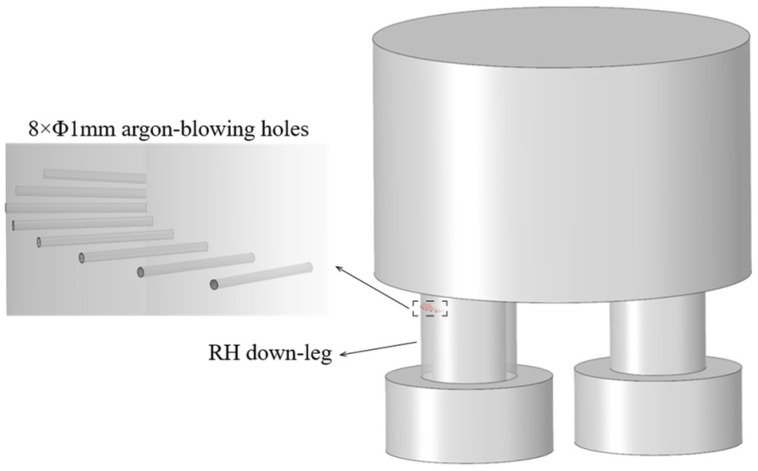
RH vacuum chamber for DLAI.

Figure 1 illustrates the customized RH vacuum chamber equipped with argon blowing holes on the down-leg. Compared to a conventional vacuum chamber, the test chamber differs primarily in the addition of a row of eight argon-blowing holes (each with an inner diameter of 1 mm) located on the side of the upper part of the down-leg, away from the up-leg. During the experiment, the total argon flow rate through the blowing holes can be monitored and adjusted via a computer in the central control room, with a range of 0~2000 L/min.

### 2.2. Method of Cold Steel Plate Dipping

**Figure 2:**
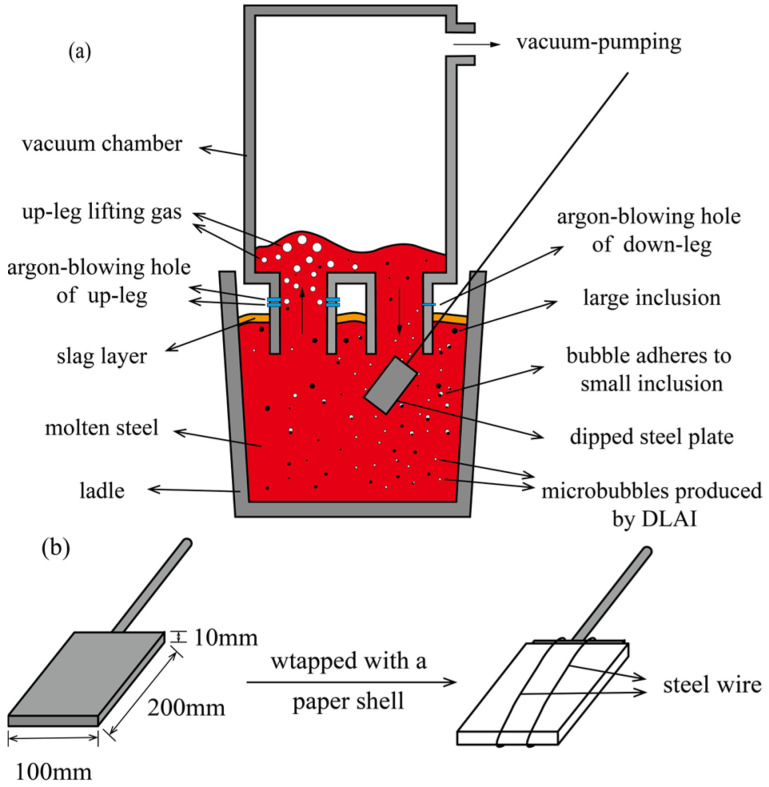
Method of ‘cold steel plate dipping’, (**a**) sampling position, (**b**) steel plate with the paper shell.

This experiment employed the ‘cold steel plate dipping’ method developed by Liu Jianhua et al. [20] to capture bubbles in molten steel during actual production. The procedure is as follows: after increasing the gas flow rate of the down-leg to the experimental level and maintaining it for 5 min, a steel plate with dimensions of 200 mm × 100 mm × 10 mm is inserted near the bottom of the down-leg and left for approximately 10 s, as shown in Figure 2a. The steel plate should be wrapped with a slag-separating paper shell to prevent the plate from being coated and adhered by the high-viscosity refining slag, and the paper shell is tied up with steel wire, as shown in Figure 2b.

Heat transfer caused the surrounding molten steel to rapidly solidify and adhere to the surface of the cold steel plate, forming a solidified steel shell. Original bubbles, inclusions, and other constituents in the solidified molten steel are also entrapped in the steel shell. The entrapped bubbles and inclusions are subsequently analyzed.

### 2.3. Method of Bubble Characteristics Analysis

**Figure 3:**
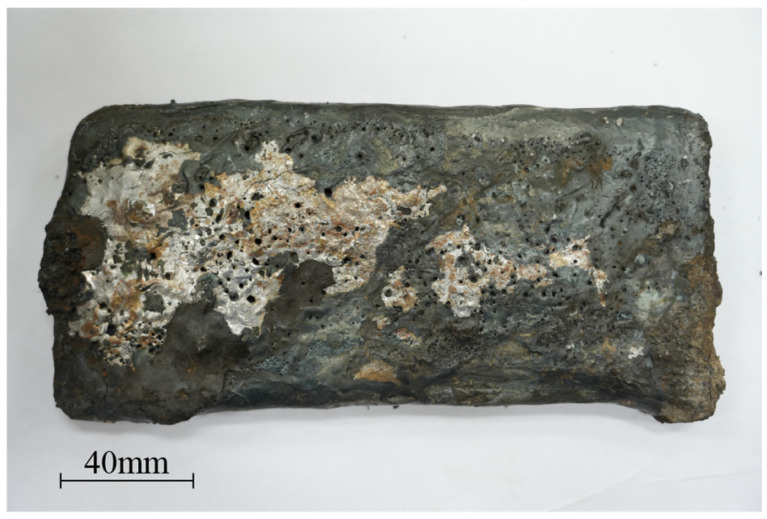
Normal temperature morphology of dipped steel plate.

The molten steel and bubbles near the bottom of the RH down-leg are captured by the dipped steel plate. The morphology of the steel plate after cooling is shown in Figure 3. There are a large number of circular holes with a diameter of 0.5 ~ 5 mm on the surface. It is observed that the forced decarburization generates substantial foaming slag. Since the steel plate will pass through the slag layer, it is speculated that the main source of the surface pores is the bubbles in the foaming slag. Therefore, in order to avoid the influence of foaming slag on bubble analysis, the surface layer of the sample should be overlooked.

**Figure 4:**
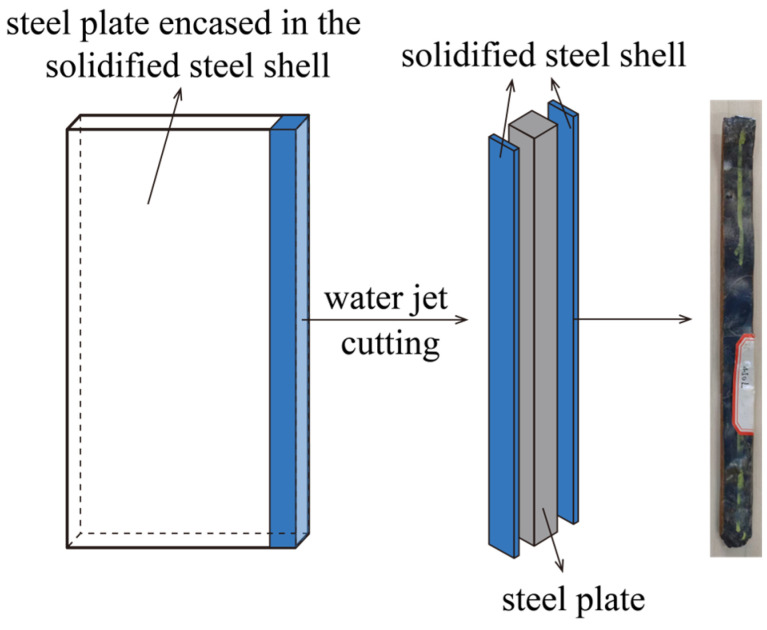
The sample of solidified steel for CT detection.

A specimen measuring 4 mm × 20 mm × 200 mm is sectioned along the longitudinal axis of the sample using a water jet cutting machine, as shown in Figure 4. Industrial computed tomography (CT) is utilized to detect internal defects within the specimen. Bubble characteristics—including size, morphology, and sphericity—are subsequently identified and quantified.

**Figure 5:**
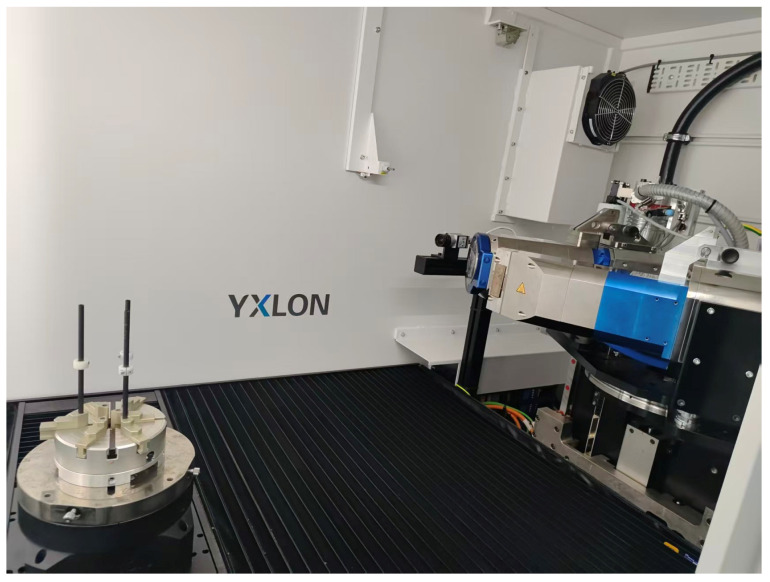
The CTYXLON FF35 industrial CT.

The instrument employed is the YXLON FF35 high-resolution industrial CT system (Comet AG, Flamatt, Switzerland), as shown in Figure 5. Its principle involves utilizing a micro-focus X-ray source to emit cone-shaped X-rays that penetrate the object and are projected onto the detector. Simultaneously, the sample rotates 360 degrees relative to the X-ray source and detector to acquire thousands of X-ray attenuation images. Subsequently, a computed tomography reconstruction algorithm is employed to generate a three-dimensional model of the sample. The CT experimental parameters are shown in Table 1.

**Figure 6:**
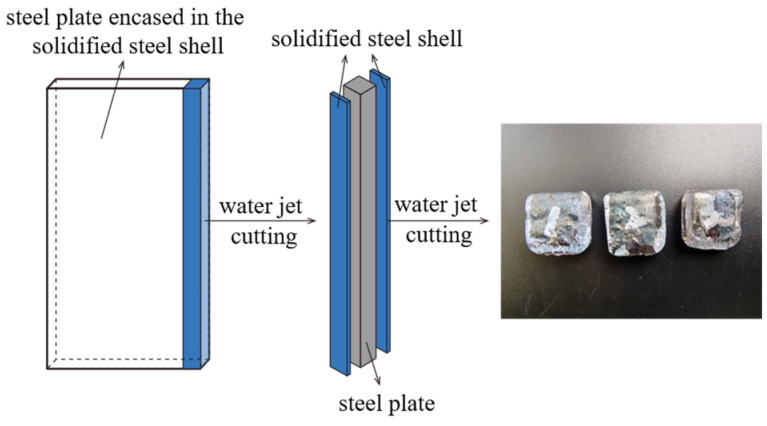
Samples for electron microscope observation.

In addition to CT detection, the ASPEX Explorer scanning electron microscope (FEI Company, Hillsboro, OR, USA) and the Zeiss Sigma 500 field emission scanning electron microscope (ZEISS Company, Oberkochen, Germany) are used to observe the microtopography of bubbles. Several specimens of 20 mm × 20 mm × 10 mm are obtained by using a water jet to cut the steel plate, as shown in Figure 6. The specimens are sequentially polished with abrasive papers of grit sizes 100#, 200#, 400#, and 800#, followed by polishing with a polishing cloth and 2.5 μm polishing paste. The polished surfaces are examined using the SEM and EDS. Among them, the operating voltage of ASPEX is 15 kV, and the vacuum degree is 3 × 10^−4^ Pa; the operating voltage of Zeiss SEM is 15 kV, and the vacuum degree is 10^−5^ Pa.

### 2.4. Method of Industrial Experiment

The gas blowing flow rates for the experimental group are specified in Table 2. Once RH decarburization is complete and aluminum pellets are added to initiate deoxidation, the gas flow rate of experimental groups is increased to the experimental value and maintained until the vacuum is released. After the vacuum is broken, the gas flow rate is restored to 20 L/min. The experimental group is divided into schemes 1 to 4, with argon flow rates of 100 L/min, 200 L/min, 450 L/min, and 600 L/min, respectively. The argon flow rate of the control group is 0.

To analyze the content of oxygen in molten steel, samples are collected from both the experimental and control groups as follows:

1. Samples are collected at 3, 6, and 9 min after the addition of aluminum pellets to the RH.
2. The sample is collected from the tundish pouring area when approximately 110 tons of steel remain in the ladle.
3. The third slab head from 1# continuous casting flow channel.

The steel grade used in this experiment is ultra-low carbon steel, with its composition detailed in Table 3. The selection of this steel grade is based on two reasons:

First, the ultra-low carbon steel is deoxidized by Al, resulting in the formation of numerous aluminum deoxidation products during the RH refining process. Among these, fine alumina particles tend to adhere to the outer surface of the rod and the inner wall of the SEN during continuous casting. Therefore, employing RH DLAI technology to accelerate the flotation of fine inclusions in the ladle, and improve molten steel purity serves.

Second, cold-rolled plates of ultra-low carbon steel products must meet stringent surface quality requirements. To this end, the steel plant employs a surface quality detector to assess surface defect size, quantity, location, and type on cold-rolled plates. The inclusion defect data obtained serve as direct evidence of the effectiveness of DLAI technology in inclusion control.

The production process of ultra-low carbon steel in this plant follows the BOF-RH-CC method. To minimize the impact of industrial production variability and improve comparability between the experimental group and the control group, some measures are implemented in this experiment:

1. The control and experimental groups are alternately produced under identical conditions, utilizing the same steel grade, equipment, and raw materials, with the production operated by the same team of workers.

2. Maximizing the sample sizes of both the experimental and control groups. Due to real-time scheduling adjustments on-site, experimenters are unable to be present during the production of ultra-low carbon steel, resulting in a smaller sample size for the experimental group compared to the control group. During the experiment, the experimental group produced 18 heats (2937.26 tons) of molten steel, whereas the control group produced 39 heats (7230.50 tons).

## 3. Results and Discussion

### 3.1. Mechanism of Micro Inclusions Removal by Bubbles

There have been numerous studies on the collision, adhesion, and detachment processes between bubbles and particles in fields such as flotation. According to Verrelli [25], the total probability of inclusion removal by bubbles is given by

(1) $$P=P_{c}\times P_{a}\times P_{d},$$

where $P_{c}$ is the collision probability between bubbles and inclusions, $P_{a}$ is the adhesion probability, and $P_{d}$ is the probability of non-detachment.

L. Zhang [26] reported that when the inclusion diameter is less than 10 μm, the collision duration between the bubble and the inclusion exceeds the time required for the liquid film between them to become sufficiently thin and rupture. Consequently, the inclusion tends to adhere to the bubble after collision. Therefore, for inclusions smaller than 10 μm, the adhesion probability ($P_{a}$) is assumed to be 1.

S. Zhang [27] found that when the inclusion diameter is less than 100 μm, the surface tension between the inclusion and the bubble dominates other forces. This surface tension causes the liquid interface to contract, pulling the inclusion closer to the bubble and making detachment difficult for small inclusions. Hence, for inclusions smaller than 100 μm, the probability of non-detachment ($P_{d}$) is assumed to be 1.

Therefore, for inclusions smaller than 10 μm, it can be considered that $P=P_{c}$.

According to Sutherland [28], the $P_{c}$ is calculated as Equations (2) and (3):

(2) $$P_{c}=\frac{-G}{1-G}{\left(1+\frac{d_{I}}{d_{B}}\right)}^{2}+\frac{3}{1+G}\frac{d_{I}}{d_{B}},$$

(3) $$G=\frac{u_{I}}{u_{B}},$$

where *P_c_* is the collision probability, %. G is a dimensionless number. $d_{I}$ is the diameter of inclusion, m. $d_{B}$ is the bubble diameter, m. $u_{I}$ is the inclusion velocity, m/s. $u_{B}$ is the bubble velocity, m/s.

The velocities of inclusions and bubbles are obtained by the Stokes formula, as Equation (4):

(4) $$u_{P}=\frac{1}{18}\frac{\left(\rho_{F}-\rho_{P}\right)g{d_{P}}^{2}}{\mu },$$

where $u_{P}$ is the particle velocity, m/s. $\rho_{F}$ is the density of molten steel, 7000 kg/m^3^. $\rho_{P}$ is the particle density, kg/m^3^. g is the acceleration due to gravity, 9.8 m/s^2^. $d_{P}$ is the particle diameter, m. μ is the viscosity of molten steel, 0.005 Pa·s.

**Figure 7:**
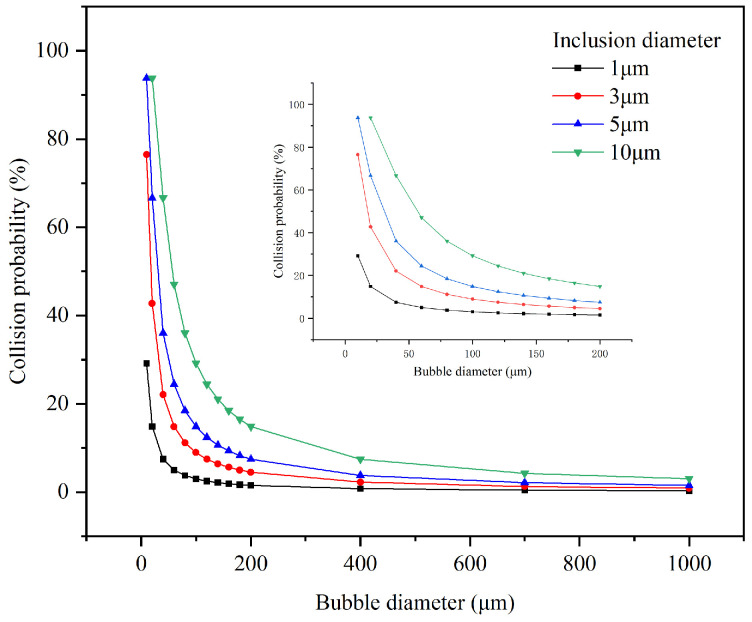
Collision probability between bubbles and tiny inclusions.

The observed bubble sizes in this experiment primarily range from 10 to 1000 μm. Consequently, the collision probability between bubbles and inclusions smaller than 10 μm is analyzed within this size range. The results are shown in Figure 7. It is evident that the collision probability between bubbles and inclusions decreases as the bubble diameter increases. Additionally, as the inclusion diameter decreases, the collision probability decreases.

For 1 μm inclusions, the collision probability with 10 μm bubbles is approximately 30% and decreases significantly as bubble size increases. The collision probability with bubbles of 60 μm diameter and larger is less than 5%, indicating a low likelihood of collision. For inclusions with diameters of 3 μm, 5 μm, and 10 μm, the collision probabilities with 60 μm bubbles are approximately 14%, 24%, and 47%, respectively.

The Sutherland model primarily addresses the interception effect of bubbles on particles in potential flow. However, during the RH refining process, the molten steel flow within the ladle is turbulent. Consequently, the influence of turbulence on bubbles and inclusions must be considered. According to studies by Shahbazi [29] and Zhao [30], turbulence exerts a relatively minor influence on the adhesion and detachment of small particles. Therefore, the effect of turbulence on the collision probability should be considered.

According to the Saffman–Turner [31] model, when bubble and inclusion sizes are relatively small, both follow turbulent micro-vortices and collide within the collision radius. The number of collisions between a single bubble and inclusions per unit time is given by

(5) $$N_{c}=n_{I}\times {\left(r_{B}+r_{I}\right)}^{3}\times {\left(\frac{8\pi \varepsilon }{15\nu }\right)}^{\frac{1}{2}},$$

where $N_{c}$ is the number of collisions between one bubble and inclusions per unit time, 1/s. $n_{I}$ is the volume number density of inclusions, 1/m^3^. $r_{B}$ is the bubble radius, m. $r_{I}$ is the inclusion radius, m. ε is the turbulent kinetic energy dissipation rate of molten steel, m^2^/s^3^. ν is the kinematic viscosity of molten steel, m^2^/s.

**Figure 8:**
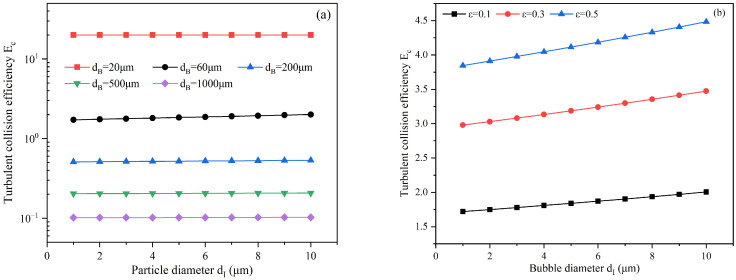
Turbulent collision efficiency of bubbles and inclusions, (**a**) ε = 0.1 m^2^/s^3^, (**b**) d_B_ = 60 μm.

Meanwhile, Nguyen [32] defined that the ideal collision number between bubbles and particles is determined by the ideal relative motion of bubbles and particles under the action of gravity, and it is described as

(6) $$N_{ci}=n_{I}\times {\pi \left(r_{B}+r_{I}\right)}^{2}\times \left(u_{B}+u_{I}\right),$$

by combining Equations (5) and (6), the collision efficiency between particles and bubbles in turbulence is obtained.

(7) $$E_{c}=\frac{N_{c}}{N_{ci}}=0.41\times \frac{r_{B}+r_{I}}{u_{B}+u_{I}}\times {\left(\frac{\varepsilon }{\nu }\right)}^{\frac{1}{2}},$$

the calculation results of $E_{c}$ are shown in Figure 8. As shown in Figure 8a, when the turbulent kinetic energy dissipation rate ε = 0.1 m^2^/s^3^, the collision efficiency increases slightly with the increase in inclusion size, while it decreases significantly with the increase in bubble size. This trend is similar to the law obtained from the Sutherland model. As indicated in Figure 8b, the collision efficiency increases significantly with the increase in the turbulent kinetic energy dissipation rate. It should be noted that turbulence can significantly enhance the relative motion between bubbles and inclusions, making their relative velocity much greater than that under the influence of gravity, which results in a collision efficiency greater than 1. In general, the turbulent environment will further increase the collision probability of bubbles and small inclusions, thereby enhancing the inclusion removal ability of bubbles.

### 3.2. Bubble Characteristics Analyzed

**Figure 9:**
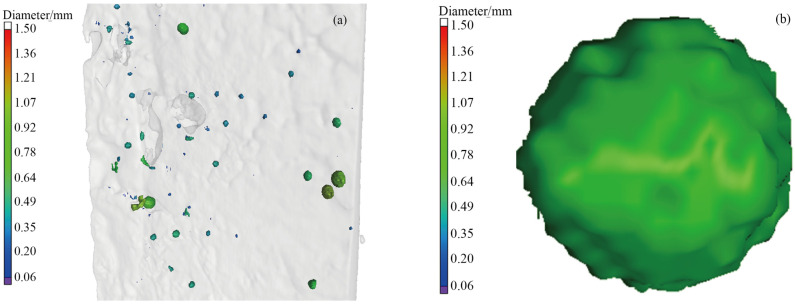
Morphology and distribution of spherical pores, (**a**) local distribution, (**b**) pore morphology.

The CT scanning revealed a large number of bubbles within the sample, with sizes ranging from 0.06 to 1.43 mm. Due to the 100 mm length of the sample, the bubbles in the three-dimensional map appear too small. Therefore, a local region is magnified, as shown in Figure 9a. Spherical pores are clearly visible, with diameters predominantly below 1 mm. The size and morphology of a single ellipsoidal pore is determined through re-magnification, as shown in Figure 9b.

**Figure 10:**
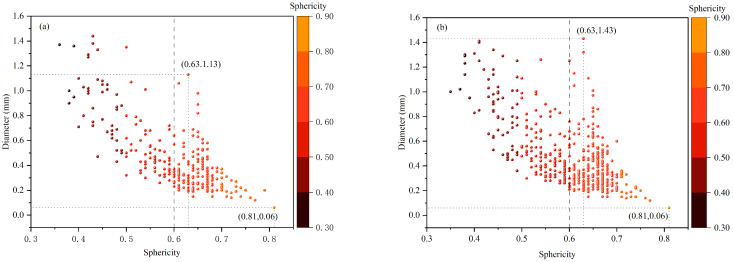
The diameter and sphericity of feature points detected by CT, (**a**) the lower part of the sample, (**b**) the upper part of the sample.

The relationship between pore sphericity and diameter in the upper and lower samples is analyzed. Since the bubble of diameter < 3 mm is spherical [33], features with sphericity of at least 0.6 are classified as bubbles. The results are shown in Figure 10.

Figure 10 indicates that smaller pore diameters are associated with higher sphericity. This is because actual bubbles are not perfect spheres, and smaller bubbles tend to approximate spherical shape due to surface tension effects. In the lower sample, the maximum bubble diameter is 1.13 mm, while the minimum is 0.06 mm. In the upper sample, the maximum bubble diameter is 1.43 mm, with a minimum of 0.06 mm.

**Figure 11:**
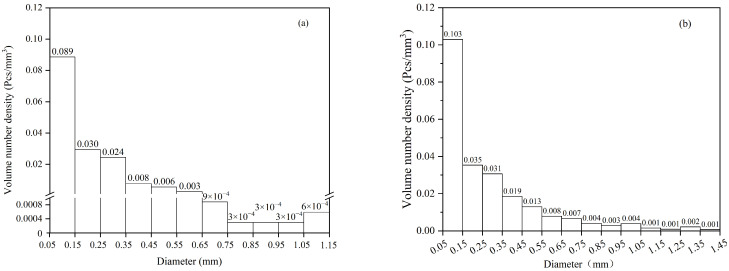
The volume number density of bubbles of different sizes, (**a**) the lower part of the sample, (**b**) the upper part of the sample.

Based on the CT detection, the total volume of the upper sample is 4.7 × 10^3^ mm^3^, while that of the lower sample is 3.4 × 10^3^ mm^3^. The volume number density of bubbles with varying diameters in the sample is calculated, as shown in Figure 11. The total bubble number density in the lower sample is 0.1606/mm^3^, with bubbles of diameters between 0.05 and 0.55 mm having a density of 0.1556/mm^3^, accounting for 96.88%. The average bubble diameter in this sample is approximately 0.26 mm. In the upper sample, the total bubble number density is 0.2306/mm^3^, with bubbles of diameters between 0.05 and 0.55 mm constituting approximately 86.9%, and the average diameter is 0.27 mm. Generally, the most common bubble diameter is 0.06 mm, with a corresponding number density of about 0.1/mm^3^.

It should be noted that the bubble size distribution is expected to approximately follow a log-normal distribution [34]. However, the number of bubbles larger than 0.06 mm detected in this experiment increases as the diameter decreases, indicating the presence of a substantial number of bubbles smaller than 0.06 mm. Subsequent electron microscope observations of the samples support this inference.

Overall, the bubble sizes detected by the DLAI are small, with the maximum size not exceeding 1.5 mm. According to the mechanism of bubble-capturing inclusions, the small-sized bubbles generated by DLAI are conducive to the inclusion removal.

**Figure 12:**
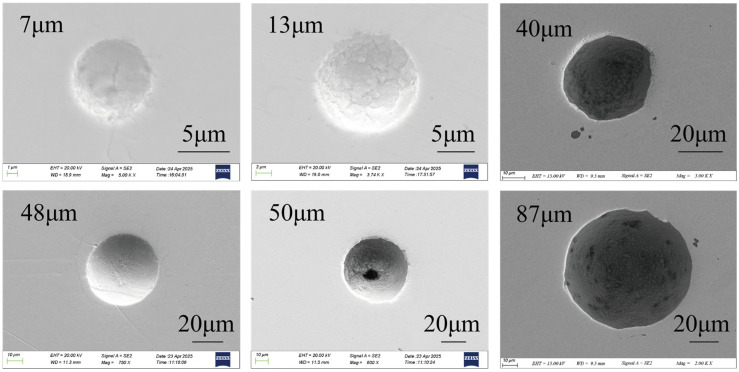
Bubble morphology and size.

In addition to the CT scanning results, SEM observation also reveals small-sized bubbles. Figure 12 presents an image of the bubbles. It is found that the majority of bubbles have diameters ranging from 40 to 50 μm, consistent with the CT test results. The maximum observed bubble diameter is 87 μm, while the minimum is 7 μm.

**Figure 13:**
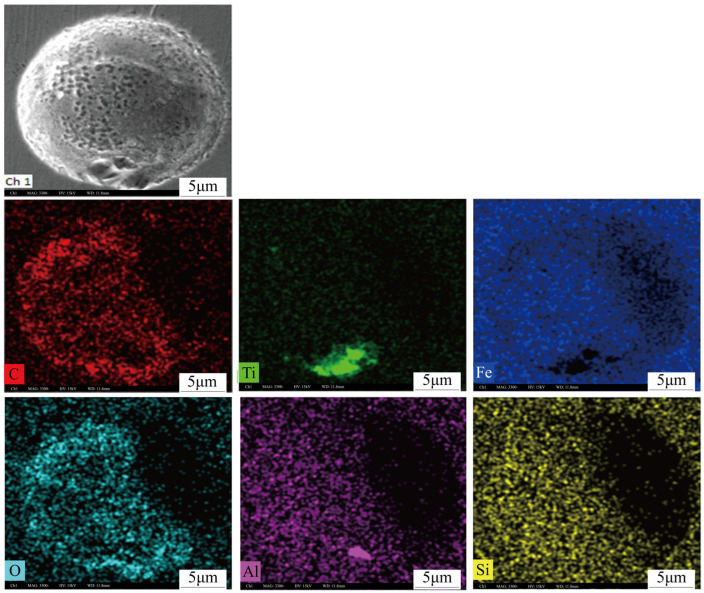
The bubble and inclusion composition.

Scanning electron microscopy and energy spectrum analysis reveal that certain bubbles adhered to inclusions. As shown in Figure 13, an Al-Ti-O inclusion approximately 8 μm in size is observed at the bottom of a bubble with a diameter of about 25 μm.

### 3.3. Effect of DLAI on Molten Steel Deoxidation

**Figure 14:**
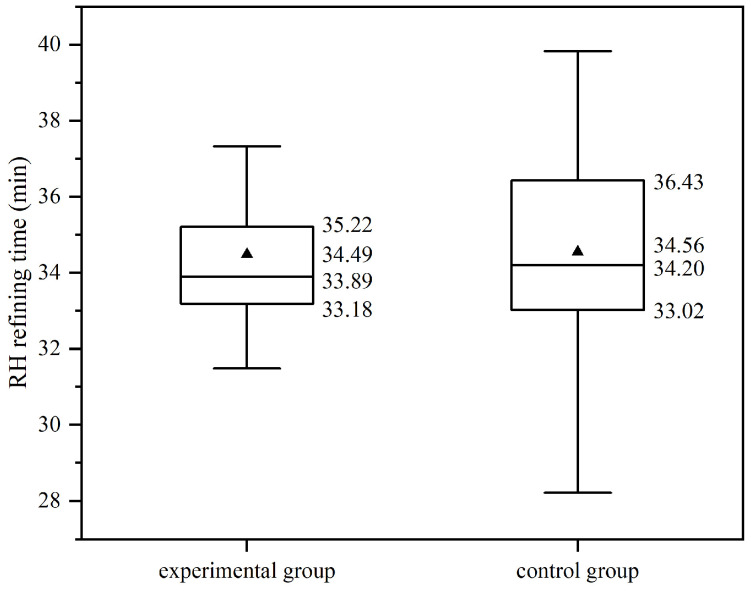
The RH refining time of the experimental and control groups.

The effect of DLAI on steel oxygen content is investigated by analyzing RH refining production data. As shown in Figure 14, the distribution of RH refining times in the experimental group is more concentrated compared to the control group. Specifically, the refining times for the experimental heats mainly range from 33.18 to 35.22 min, whereas those for the control group heats mainly range from 33.02 to 36.43 min. Additionally, the average refining times (the triangle in Figure 14) are similar, at 34.49 min for the experimental group and 34.56 min for the control group. The median values are also comparable, at 33.89 min for the experimental group and 34.20 min for the control group.

The standard error of the experimental group is 0.66 min, which is less than 0.1 of the mean value, and the 95% confidence interval of the mean value is (33.10 min, 35.88 min). The standard error of the control group is 0.41 min, which is less than 0.1 of the mean value, and the 95% confidence interval of the mean value is (33.74 min, 35.38 min). The mean absolute deviations of the experimental group and the control group are 1.99 min and 1.97 min, respectively, with the experimental group being 101% of the control group, indicating that the degree of dispersion of the two groups is basically the same.

Overall, these results indicate that the DLAI technology does not significantly affect the RH refining duration.

**Figure 15:**
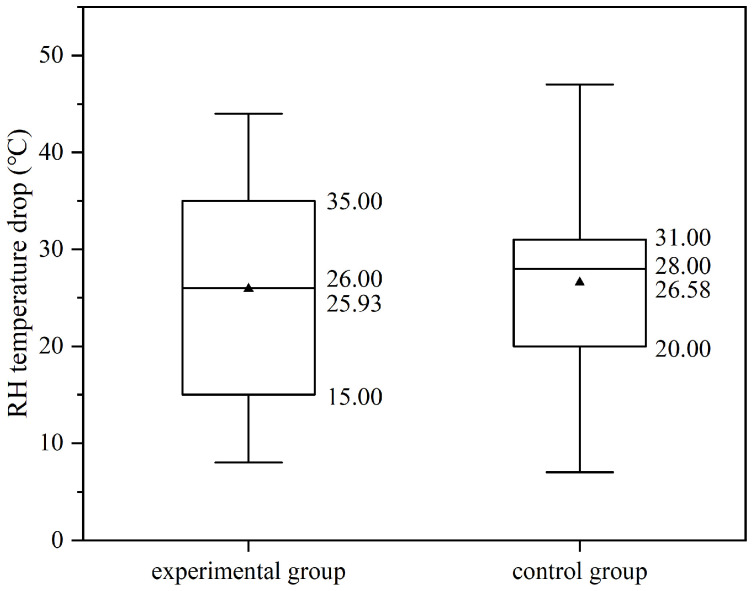
RH temperature drop of the experimental and control groups.

The DLAI technology blows low-temperature argon into the molten steel at a significant flow rate. Therefore, it is essential to analyze its impact on the temperature drop of molten steel in the RH process. As shown in Figure 15, the distribution of RH temperature drops for both the experimental and control groups is similar. The outlet temperature drop for the experimental group primarily ranges from 15.00 to 35.00 °C, whereas that for the control group mainly falls within 20.00 to 31.00 °C. Their average temperature drops are approximately the same, at 25.93 °C and 26.58 °C, respectively. Similarly, the median values are close, at 26.00 °C and 28.00 °C, respectively. Overall, the distribution range, mean, and median of the temperature drops in the experimental group are consistent with those in the control group, with differences of approximately 1–2 °C.

The standard error of the experimental group is 2.96 °C. The ratio of the standard error to the mean value is less than 0.3, and the 95% confidence interval of the mean value is (19.59 °C, 32.27 °C). The standard error of the control group is 1.55 °C. The ratio of the standard error to the mean value is less than 0.1, and the 95% confidence interval of the mean value is (23.43 °C, 29.73 °C). The mean absolute deviations of the temperature drop in the experimental group and the control group are 9.40 °C and 7.46 °C, respectively. The experimental group is 126% of the control group, indicating that the dispersion degree of the temperature drop in the experimental group is relatively larger.

It can be concluded that the DLAI technology has no significant effect on the temperature drop in the RH process.

In summary, the refining duration and temperature decline of the heats in both the experimental and control groups are comparable. It can be concluded that the deoxidation duration and reaction temperature during the refining process of molten steel are similar in both groups. Therefore, the effect of DLAI on the deoxidation reaction can be demonstrated by comparing the oxygen activity at the start and end of the RH refining.

**Figure 16:**
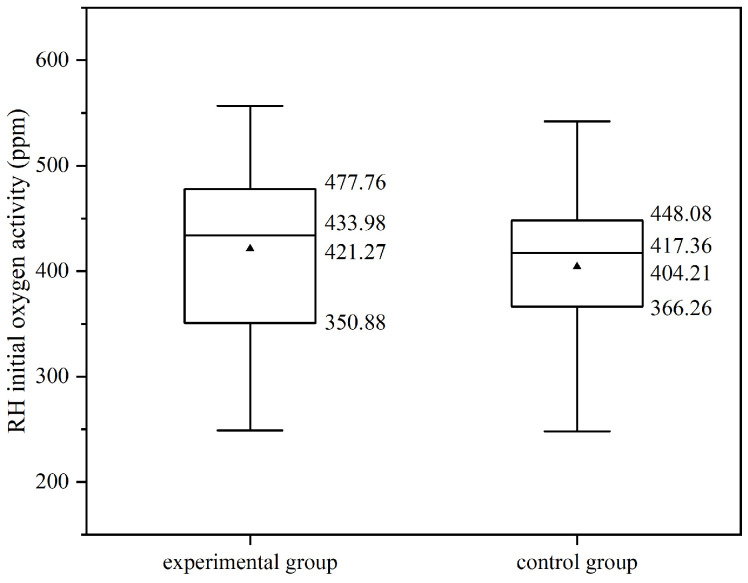
Initial RH oxygen activity of experimental and control groups.

According to Figure 16, the distribution of oxygen activity at the RH start in both the experimental and control groups is similar. The initial oxygen activity in the experimental group primarily ranges from 350.88 to 477.76 ppm, while in the control group it ranges from 366.26 to 448.08 ppm. Additionally, the average initial oxygen activity in the experimental group (421.27 ppm) is slightly higher than that in the control group (404.21 ppm). Similarly, the median inlet oxygen activity in the experimental group (434.0 ppm) is marginally higher than in the control group (417.4 ppm).

The standard error of the initial oxygen activity in the experimental group is 21.09 ppm, which is less than 0.1 of the mean value, and the 95% confidence interval of the mean value is (376.77 ppm, 465.77 ppm). The standard error of the initial oxygen activity in the control group is 15.18 ppm, which is less than 0.1 of the mean value, and the 95% confidence interval of the mean value is (373.51 ppm, 434.91 ppm). The mean absolute deviations of the experimental group and the control group are 72.00 ppm and 66.15 ppm, respectively, with the experimental group being 109% of the control group, indicating a small difference in the degree of dispersion between the two groups.

Overall, the initial oxygen activity in the experimental group is marginally higher than in the control group.

**Figure 17:**
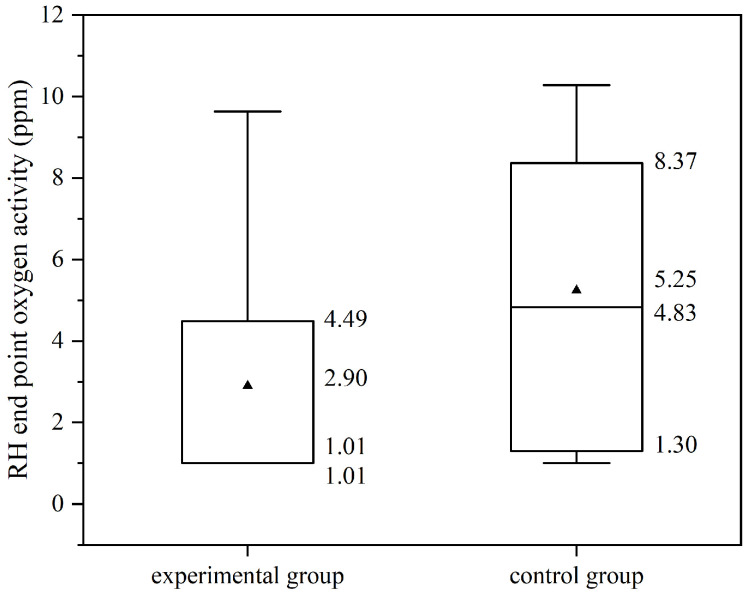
RH endpoint oxygen activity of experimental and control groups.

Figure 17 illustrates the oxygen activity at the end of the RH process for both experimental and control groups. Following deoxidation with aluminum addition, the oxygen activity in the molten steel is generally below 10 ppm. The data distribution range of the experimental groups is generally narrower than that of the non-experimental groups. The end oxygen activity of the experimental groups predominantly ranges from 1.01 to 4.49 ppm, with a median of 1.01 ppm and an average of 2.90 ppm. In contrast, the control groups primarily range from 1.30 to 8.37 ppm, with a median of 4.83 ppm and an average of 5.25 ppm. Overall, the end oxygen activity in the experimental groups is reduced by an average of 2.35 ppm compared to the non-experimental groups, representing an approximate 50% decrease.

The standard error of the endpoint oxygen activity in the experimental group is 0.73 ppm, the ratio of the standard error to the mean is less than 0.3, and the 95% confidence interval of the mean is (1.35 ppm, 4.45 ppm). The standard error of the control group is 0.48 ppm, the ratio of the standard error to the mean is less than 0.1, and the 95% confidence interval is (4.28 ppm, 6.21 ppm). The mean absolute deviations of the experimental group and the control group are 2.53 ppm and 2.46 ppm, respectively. The mean absolute deviation of the experimental group is 103% of that of the control group, and the difference between the two is small.

By comparison, it is credible that the endpoint oxygen activity of the experimental group is lower than that of the control group.

**Figure 18:**
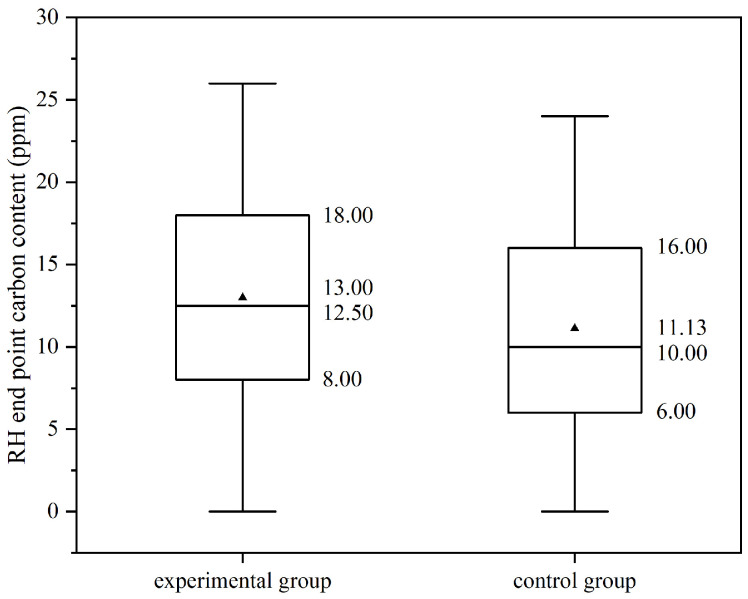
RH endpoint carbon content of experimental and control groups.

A decrease in oxygen activity at the end of RH during the experimental groups, as indicated by the carbon–oxygen balance, leads to an increase in carbon content. Figure 18 illustrates the carbon content at the end of RH. The end carbon content in the experimental groups predominantly ranges from 8.00 ppm to 18.00 ppm, with a mean of 13.00 ppm and a median of 12.5 ppm. In contrast, the control groups exhibit a range from 6.00 ppm to 16.00 ppm, with a mean of 11.13 ppm and a median of 10.00 ppm.

The standard error of the endpoint carbon activity in the experimental group is 1.58 ppm, which is less than 0.3 of the mean, and the 95% confidence interval of the mean is (9.67 ppm, 16.33 ppm). The standard error of the endpoint carbon activity in the control group is 0.95 ppm, which is less than 0.1 of the mean, and the 95% confidence interval of the mean is (9.20 ppm, 13.06 ppm). The mean absolute deviations of the experimental group and the control group are 5.44 ppm and 4.86 ppm, respectively. The experimental group is 111% of the control group, and the difference between the two is small.

It can be found that the RH endpoint carbon content of the experimental group is slightly higher than that of the control group.

Overall, the implementation of DLAI technology does not significantly affect the RH refining duration or the temperature decline. Within the same refining duration, the molten steel in the experimental group exhibits lower oxygen activity and higher carbon content at the conclusion of RH refining. This suggests that DLAI enhances oxidation reactions within the molten pool, likely because the bubble-driven removal of deoxidation products accelerates the deoxidation process.

**Figure 19:**
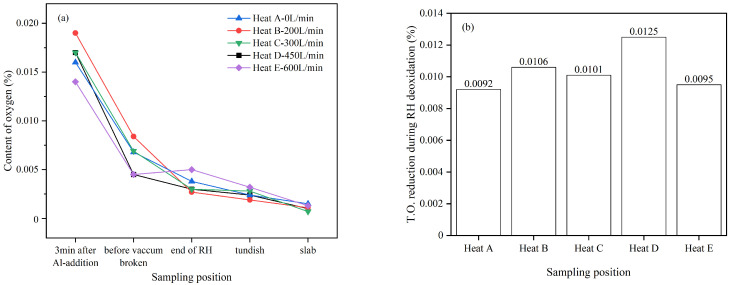
The T.O. statistics, (**a**) The T.O. from RH deoxidation to casting, (**b**) The T.O. reduction during RH deoxidation.

In addition to the industrial data, the T.O. content in steel during the process from RH deoxidation to continuous casting is analyzed, as shown in Figure 19a. The results indicate that the oxygen content in the molten steel for each heat exhibits a decreasing trend as the process progresses.

The reduction in T.O. in molten steel during the RH deoxidation process is compared, as shown in Figure 19b. It is found that the oxygen reduction in the experimental groups (heats B~E) is all higher than that of the control group (heat A). Among them, heat D with an argon injection flow rate of 450 L/min had the highest oxygen reduction, reaching 0.0125%. This suggests that DLAI technology contributes to removing deoxidation products from the molten steel.

### 3.4. Effect of DLAI on Cold-Rolled Sheet Surface Quality

During the rolling process, the surface quality of the rolled plate may deteriorate due to defects caused by inclusions, scratches, and embedded foreign matter. The mass of the rolled plate removed due to defects is termed the downgrading weight, and the ratio of this weight to the slab’s total weight is the downgrading rate. The downgrading rate attributable to inclusion defects is termed the inclusion downgrading rate. This parameter is crucial for directly assessing the level of inclusion control in the slab. By comparing the inclusion downgrading rates of the control and experimental groups, the effect of the DLAI technology for controlling inclusions can be demonstrated.

**Figure 20:**
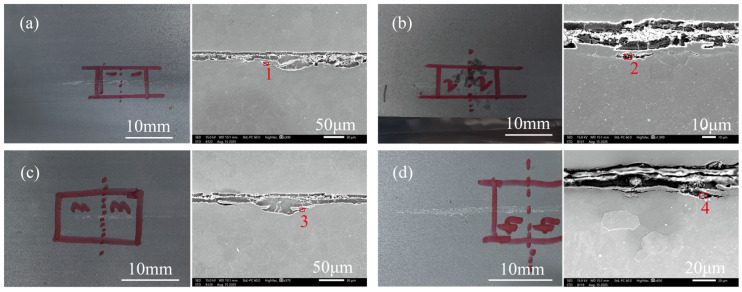
Macroscopic and microscopic morphologies of surface defects in cold-rolled plates, (**a**) Cold-rolled sheet No. 1, (**b**) Cold-rolled sheet No. 2, (**c**) Cold-rolled sheet No. 3, (**d**) Cold-rolled sheet No. 4.

To verify the accuracy of the surface quality detector in identifying inclusion defects on the surface of cold-rolled sheets, four cold-rolled sheets determined to have inclusion defects were obtained, and electron microscope scanning and EDS were used on the defective areas, as shown in Figure 20. According to the energy spectrum components shown in Table 4, it is shown that the surface defects of cold-rolled sheets No. 1, No. 3, and No. 4 are caused by alumina inclusions, and the defect of No. 2 is caused by slag entrainment. This indicates that the identification of inclusion defects by the surface inspection instrument is reliable.

The statistical results of the total weight, total downgrading weight, and total downgrading rate of the control group and the experimental group are shown in Table 5. The downgrading rate of cold-rolled sheets in the experimental group was 5.07% lower than that in the control group, with a significant gap. However, the total weight of the control group is approximately 2.46 times that of the experimental group, indicating a moderate sample size imbalance. Statistical analysis is required to determine whether this imbalance has an impact on the results.

A significance test for differences between the two groups of data is conducted; a two-sample independent proportion two-tailed Z-test is used to verify whether there is a statistical difference in the total downgrading rates between the two groups. The test hypotheses are established as follows: the null hypothesis H_0_: there is no difference in the total downgrading rates between the two groups, and the alternative hypothesis H_1_: there is a difference in the total defect rates between the two groups, with a significance level α = 0.05.

The merged defect rate is calculated as:

$$\hat{P}=\frac{x_{1}+x_{2}}{n_{1}+n_{2}}=11.16\%,$$

where $x_{1}$ is the downgrading weight of the control group, $x_{2}$ is the downgrading weight of the experimental group, $n_{1}$ is the total weight of the control group, $n_{2}$ is the total weight of the experimental group.

The Z statistics are calculated as:

$$Z=\frac{P_{1}-P_{2}}{\sqrt{\hat{P}(1-\hat{P})(\frac{1}{n_{1}}+\frac{1}{n_{2}})}}=7.37,$$

where $P_{1}$ is the downgrading rate of the control group, $P_{2}$ is the downgrading rate of the experimental group.

Critical values for a two-tailed test of the standard normal distribution Z_0.05/2_ = 1.96, and in this study, Z = 7.37 > 1.96, even Z > 3.29. Therefore, the probability of the null hypothesis H_0_ occurring is less than 0.001, so the alternative hypothesis H_1_ should be accepted, indicating that the difference in the total downgrading rate of cold-rolled sheets between the control group and the experimental group is extremely statistically significant. Therefore, although the samples of the experimental group and the control group are unbalanced, the results are not significantly affected.

The DLAI technology adopted in this study aims to reduce the amount of alumina in molten steel, thereby reducing the surface defects of cold-rolled sheets caused by alumina. However, the detection results of the surface quality detector do not specifically distinguish whether the defects are caused by slag or alumina. Therefore, when discussing the impact of DLAI technology on the defects of cold-rolled sheets, it is necessary to compare and explain the occurrence probability of slag entrainment defects between the experimental group and the control group.

The direct cause of slag entrainment is the fluctuation of the mold liquid level. The factory monitors the mold liquid level fluctuation in real-time. According to the amplitude and frequency of the liquid level fluctuation during the continuous casting process, the slabs are divided into four grades: a, b, c, and d, among which grade a has the smallest liquid level fluctuation and grade d has the largest. According to on-site data as shown in Table 6, among the slabs in the experimental group, the proportions of grade a, b, c, and d slabs are 9.47%, 44.89%, 3.26%, and 42.39%, respectively. Among the slabs produced in the control group, the proportions are 9.75%, 48.59%, 1.45%, and 40.22%, respectively. Therefore, the proportions of grade a and b slabs in the experimental group are slightly lower, while those of grade c and d are slightly higher, but the overall difference is not significant. Under the premise of a large sample size, it can be considered that the probability of slag entrapment defects occurring in the two groups of cold-rolled sheets is basically the same. Therefore, it can be considered that the reason for the reduction in inclusion defects in the experimental group is the reduction in alumina in the molten steel.

For the comparison and analysis, slabs from both the control and experimental groups are classified into five grades: a, b, c, d, and e. This classification is based on the fact that the expected quality and cleanliness of grade a slabs are the highest and grade e the lowest. Different grades of slabs have corresponding detection standards for inclusion defects during the rolling process. Therefore, it is necessary to calculate the inclusion downgrading rates separately for slabs of each grade.

**Figure 21:**
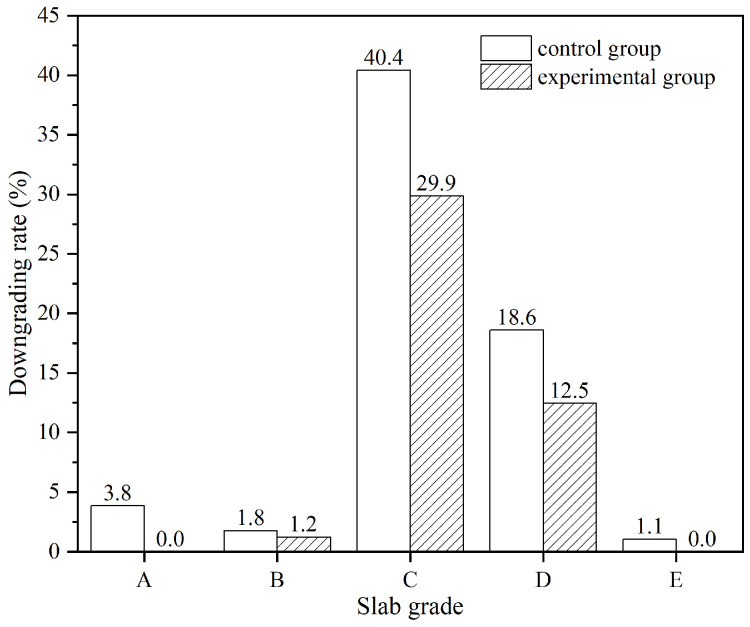
Inclusion downgrading rate of experimental and control groups.

The downgrading rates of slabs in both experimental and control groups are illustrated in Figure 21. It is indicated that the downgrading rates of slabs in the experimental group are consistently lower than the control group. Among these, the most significant reduction is observed in grade c slabs, with a downgrading rate decreasing by 10.5 percentage points. For grade a slabs, the downgrading rate in the experimental group decreased by 3.8%, effectively achieving ‘zero defect’ status. Compared to the control group, the total downgrading rate of the experimental group decreased by 5.07%.

**Figure 22:**
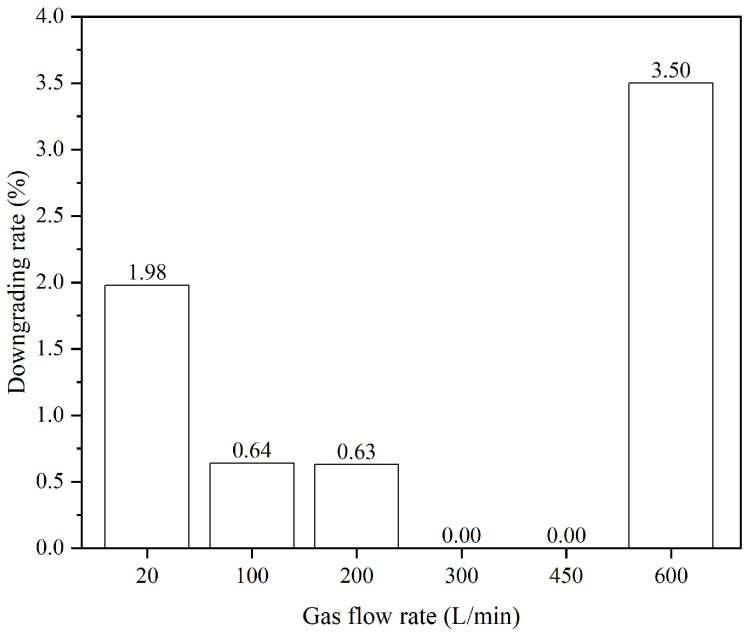
Inclusion downgrading rate of the experimental group under varying argon flow rates.

The statistical comparison of the inclusion degrading rate produced under different argon blowing flow rates is conducted, as illustrated in Figure 22. The results show that the inclusion downgrading rate initially decreases and then increases with increasing argon flow rate, reaching its minimum at flow rates of 300 L/min and 450 L/min. This phenomenon can be attributed to the fact that at low argon flow rates, fewer bubbles are generated, resulting in lower adhesion and removal efficiency for inclusions. Conversely, at higher flow rates, larger bubbles are produced, which reduce the adhesion and removal efficiency for small inclusions [35,36,37], thereby diminishing the improvement of steel cleanliness. Therefore, argon flow rates of 300 L/min and 450 L/min are considered more appropriate for the downcomer.

Overall, DLAI technology significantly enhanced the effectiveness of inclusion removal in IF steel, with optimal results observed at gas flow rates between 300 and 450 L/min.

## 4. Conclusions

The down-leg argon injection technology is applied in the industrial production of IF steel. Samples of micro argon bubbles are obtained by the ‘cold steel plate dipping’ method, and the characteristics of bubbles are analyzed. Combined with the bubble metallurgy mechanism and the results of the industrial experiment, the effect of the down-leg argon injection technology on inclusion removal is analyzed. The conclusions are drawn as follows:

1. For inclusions with a diameter of 1~10 μm, the probability of inclusion removal by bubbles increases with the decrease in bubble diameter and increase in inclusion diameter. And the turbulent fluid will enhance the inclusion removal ability of bubbles.

2. The minimum bubble size observed in the samples is 7 μm, while the maximum reaches 1.43 mm. Bubbles with a diameter of approximately 60 μm are the most numerous, and the total volume density of bubbles exceeded 0.3 mm^−3^. The bubbles can capture inclusions smaller than 10 μm.

3. After implementing the DLAI technology, the RH refining duration and out-station temperature showed no significant change. However, the reduction in T.O. in the molten steel during the RH deoxidation process is significantly increased.

4. After implementing the DLAI technology, the surface inclusion defects in full-grade cold-rolled sheets are reduced. Among them, the cold-rolled sheets with the highest surface quality requirements achieve ‘zero defect’ status. Additionally, when the argon blowing flow rate is 300 L/min and 450 L/min, the number of inclusion defects in the cold-rolled sheet is the smallest.

## Figures and Tables

**Table 1:** CT experimental parameters.

Resolution/μm	Voltage/kV	Electric Current/μA	Integration Time/s	Scanning Time/min	Exposure Time/ms
60	180	110	0.43	20	400

**Table 2:** Setting of Experimental Gas Blowing Flow Rate.

Experimental Group	Experimental Flow Rate (L/min)
Scheme 1	100
Scheme 2	200
Scheme 3	450
Scheme 4	600

**Table 3:** Composition of IF steel for the experiment.

C	Si	Mn	P	S	Als	Ti	N
≤0.0020	≤0.010	0.05~0.08	≤0.010	≤0.009	0.020~0.050	0.065~0.085	≤0.0030

**Table 4:** Chemical composition of the defective part.

NO.	O	Na	Mg	Al	Si	S	K	Ca	Ti	Cr	Fe
1	45.98	—	—	50.43	—	—	—	—	—	—	3.58
2	8.40	0.40	1.04	2.08	2.07	—	0.26	0.50	—	—	85.25
3	41.86	—	—	53.15	—	—	—	—	1.15	0.36	3.48
4	7.56	—	—	8.50	—	—	—	—	—	8.33	76.60

**Table 5:** Total weight and total downgrading rate of cold-rolled sheets.

Group	Total Weight/t	Downgrading Weight/t	Downgrading Rate/%
Control group	7230.50	912.45	12.62
Experimental group	2937.26	221.73	7.55

**Table 6:** Proportion of slabs at different grades.

Slab Grade	Control Group	Experimental Group
a	9.47%	9.75%
b	44.89%	48.59%
c	3.26%	1.45%
d	42.39%	40.22%

## Data Availability

The original contributions presented in this study are included in the article. Further inquiries can be directed to the corresponding author.
